# The Complete Genome Sequence of *Mycoplasma bovis* Strain Hubei-1

**DOI:** 10.1371/journal.pone.0020999

**Published:** 2011-06-22

**Authors:** Yuan Li, Huajun Zheng, Yang Liu, Yanwei Jiang, Jiuqing Xin, Wei Chen, Zhiqiang Song

**Affiliations:** 1 National Contagious Bovine Pleuropneumonia Reference Laboratory, Division of Bacterial Diseases, State Key Laboratory of Veterinary Biotechnology, Harbin Veterinary Research Institute, The Chinese Academy of Agricultural Sciences (CAAS), Harbin, China; 2 Shanghai-MOST Key Laboratory of Health and Disease Genomics, Chinese National Human Genome Center at Shanghai, Shanghai, China; The University of Hong Kong, Hong Kong

## Abstract

Infection by *Mycoplasma bovis* (*M. bovis*) can induce diseases, such as pneumonia and otitis media in young calves and mastitis and arthritis in older animals. Here, we report the finished and annotated genome sequence of *M. bovis* strain Hubei-1, a strain isolated in 2008 that caused calf pneumonia on a Chinese farm. The genome of *M. bovis* strain Hubei-1 contains a single circular chromosome of 953,114 bp with a 29.37% GC content. We identified 803 open reading frames (ORFs) that occupy 89.5% of the genome. While 34 ORFs were Hubei-1 specific, 662 ORFs had orthologs in the *M. bovis* type strain PG45 genome. Genome analysis validated lateral gene transfer between *M. bovis* and the *Mycoplasma mycoides* subspecies *mycoides*, while phylogenetic analysis found that the closest *M. bovis* neighbor is *Mycoplasma agalactiae*. Glycerol may be the main carbon and energy source of *M. bovis*, and most of the biosynthesis pathways were incomplete. We report that 47 lipoproteins, 12 extracellular proteins and 18 transmembrane proteins are phase-variable and may help *M. bovis* escape the immune response. Besides lipoproteins and phase-variable proteins, genomic analysis found two possible pathogenicity islands, which consist of four genes and 11 genes each, and several other virulence factors including hemolysin, lipoate protein ligase, dihydrolipoamide dehydrogenase, extracellular cysteine protease and 5′-nucleotidase.

## Introduction

*Mycoplasma bovis* (*M. bovis*), first isolated in 1961 from a severe case of mastitis in cattle, can cause diseases, such as pneumonia, otitis media in young calves and mastitis and arthritis in older animals [1]–[3]. These diseases have been designated *M. bovis*-associated diseases (MbAD) [4]. *M. bovis* has spread widely to all parts of the world via animal movement [5]. In recent years, *M. bovis* has become an important pathogen in young calves in Europe and North America [6], and its infection results in calf mortality, weight loss in surviving calves and a drop in milk production [7]. Clinical diseases caused by *M. bovis* tend to be chronic, debilitating and unresponsive to antimicrobial therapy. For animal welfare, the veterinarian can provide only limited relief for calves subject to MbAD [8]–[9].

In addition to MbAD, contagious bovine pleuropneumonia (CBPP) is another cattle respiratory system infectious disease that is caused by *Mycoplasma mycoides* subspecies *mycoides* with a small colony biotype (MmmSC), and it exhibits similar clinical symptoms and pathological anatomy as MbAD. However, the damage caused by CBPP is more severe than by MbAD, and CBPP has been listed as a contagious disease that must be reported to World Organization for Animal Health (OIE). It is difficult to distinguish CBPP from MbAD because there is serological cross-reactivity between the two diseases. *M. bovis* has now become the most important *Mycoplasma* species in cattle in countries without CBPP.

*M. bovis* is a member of the Mollicutes class and belongs to the genus *Mycoplasma*. Although more than 20 *Mycoplasma* genome sequences have been published, the *M. bovis* genome was not published until 2010. In recent years, some genes have been discovered to exist in all *M. bovis* strains including the p48 gene [10], the p81 gene [11] and the p40 gene [12], while the p68 gene [13] is missing in some M*ycoplasma* strains. The variable membrane surface lipoproteins(Vsps) were major antigen of *M. bovis* and underwent noncoordinate phase variation [14], which has been a recent focus in *M. bovis* membrane protein research.

A severe cattle respiratory contagious disease occurred in the Hubei Province of China in 2008 that spread to over 11 Chinese provinces. The main symptoms of this disease were coughing and high fever, which led to the death of 572 out of 2,476 infected cattle. In one farm, all 62 cattle were infected and 24 died, a death ratio of 38.7%. Though these diseases show similar clinical symptoms and pathological changes with CBPP, CBPP antibody identification using the complement fixation test (CFT) reagent of National Veterinary Laboratory (LNIV) and specific PCR reactions did not produce positive results. The organisms were isolated from calf lungs with a method developed by Poumarat [15], and the isolated *Mycoplasma* strain was named Hubei-1. PCR assays targeting Hubei-1 16S rRNA demonstrated 99.5% homology with *M. bovis* type strain PG45. The *M. bovis* isolates were further confirmed by PCR and restriction endonuclease analysis (REA), which had been previously validated for the identification of *M bovis* isolates [16]. When the serum samples were tested for antibodies against *M. bovis* using a commercial kit (Biovet), positive results were obtained for almost all of the samples.

In 2004, the genomic sequence of MmmSC PG1, the pathogen responsible for CBPP, was released. In 2010, the genome of *M. bovis* type strain PG45 was reported; this was the first sequenced *M. bovis* genome, and it has provided valuable information to understand MbAD. In this paper, we report the complete genomic sequence of *M. bovis* strain Hubei-1 and compare the sequence with the genome of the *M. bovis* type strain PG45. Understanding the genetics and pathogenic mechanisms of *M. bovis* would be valuable to comprehend the difference between the genes, proteins, and nosogenesis of the two mycoplasms that cause CBPP and MbAD, and it would also be helpful in the diagnosis of the two diseases in animals.

## Results

### Genome features

**Figure 1:**
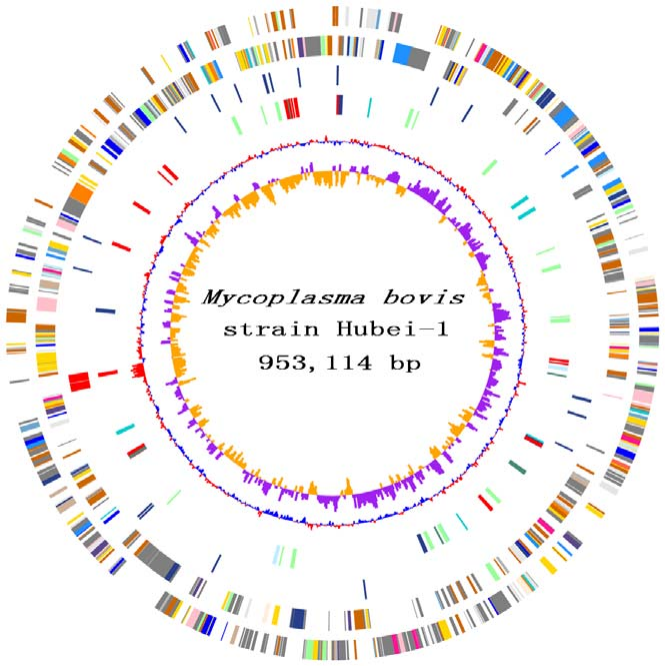
Chromosome Atlas of *Mycoplasma bovis* Strain Hubei-1. The scale is shown by the outer black circle. Moving inside, the 1^st^ and 2^nd^ circles illustrate predicted coding sequences on the plus and minus strands respectively, colored according to different functional categories. The 3^rd^ circle represent tRNAs (blue) and ribosomal RNA genes (red). The 4^th^ circle display IS elements. The 5^th^ and 6^th^ (innermost) circle represent mean centered G+C content of the genome (red-above mean, blue-below mean) and GC skew (G−C)/(G+C), respectively, calculating using a 1-kb window in steps of 500 bp.

The *M. bovis* strain Hubei-1 genome contains a single circular chromosome of 953,114 bp with a GC content of 29.37% (**Figure 1**). We identified 803 ORFs in the genome with an average length of 1,058 bp and a mean GC content of 29.76% that occupied 89.5% of the genome. Among these ORFs, 523 (65.1%) genes could be classified into Clusters of Orthologous Groups (COG) families comprising 18 functional categories (**Table 1**). Biological functions were assigned to 490 (60.9%) genes, and 30 genes encoded hypothetical proteins. The genome encodes two rRNA operon and 34 tRNAs that represent all 20 amino acids. The genome sequence data were deposited in Genbank with the accession number CP002513.

**Table 1:** Functional Categories in COG of *M.bovis*.

	Functional Category	Hubei-1	PG45	Common
C	Energy production and conversion	27	27	27
D	Cell division and chromosome partitioning	9	9	8
E	Amino acid transport and metabolism	26	25	25
F	Nucleotide transport and metabolism	20	20	20
G	Carbohydrate transport and metabolism	38	37	36
H	Coenzyme metabolism	13	13	13
I	Lipid metabolism	8	7	7
J	Translation,ribosomal structure and biogenesis	102	105	102
K	Transcription	17	18	17
L	DNA replication, recombination and repair	96	93	63
M	Cell envelope biogenesis, outer membrane	16	15	15
N	Cell motility and secretion	1	1	1
O	Posttranslational modification, protein turnover, chaperones	19	20	19
P	Inorganic ion transport and metabolism	12	12	12
Q	Secondary metabolites biosynthesis, transport and catabolism	1	1	1
R	General function prediction only	63	61	61
S	Function unknown	31	34	30
T	Signal transduction mechanisms	4	4	4
U	Intracellular trafficking and secretion	10	9	8
V	Defense mechanisms	18	15	13
-	Not in COGs	272	239	180
Total		803	765	662

As a low GC content genome, 80% of the codons have an A or a T at the wobble position in *M. bovis* resulting in a TAA stop codon in 76.3% of the genes. The three codons that encode for Arginine (i.e., CGG, CGA and CGC) ranked the lowest in the genome, occupying only 0.2% of all codons (**Table S1**), which indicates a low usage efficiency of tRNA^arg^ [ACG].

*M. bovis* strain Hubei-1 encodes 73 lipoproteins, 36 secreted proteins and 134 transmembrane proteins, indicating that 30.2% of *M. bovis* encoded proteins are associated with the extracellular environment. Accordingly, a lipoprotein signal peptidase gene (*lsp, MMB_0238*) and a prolipoprotein diacylglyceryl transferase gene (*lgt, MMB_0093*) were predicted in the genome; however, no signal peptidase I gene was found.

### IS elements

We found 26 complete insertion sequence (IS) elements in the genome including the known elements IS*Mbov1*, IS*Mbov2*, and IS*Mbov3* and three newly identified IS elements, which were designated as IS*Mbov4*, IS*Mbov5* and IS*Mbov6* (**Table S2**). IS*Mbov4* is 1,340 bp long and encodes for a 414 amino acid transposase that shows 50% identity with the transposase IS*1138* [17] and could be classified in the IS3 family. There are eight full length and eight truncated copies of IS*Mbov4* in the *M. bovis* genome that exhibit the same copy number as IS*Mbov1*.

IS*Mbov5* is 1,468 bp and encodes for a 477 amino acid transposase that belongs to the IS*4* family. There are only two complete copies of ISM*bov5* in the genome. IS*Mbov6* is 1,415 bp long, encodes a 338 amino acid transposase and belongs to the IS*30* family. There are three complete and four truncated copies of ISM*bov6* in the genome. Of note, two IS*Mbov2* elements were disrupted by IS*Mbov4* insertion, and two IS*Mbov4* elements were disrupted by the insertion of IS*Mbov3* reflecting an insertion bias of IS elements.

All of the IS elements are evenly distributed across the genome, occupying 5.4% of the *M. bovis* genome.

### Tandem repeats and phase variation

It has been reported that tandem repeats upstream or inside the *Mycoplasma* coding sequence (CDS) would cause phase-variable lipoprotein gene expression [18]–[20] because polymerase slippage would occur in tandem repeat regions during replication.

We identified 371 tandem repeat loci in the genome (**Table S3**). Here, tandem repeat refers to either a repeat unit ≥10 bp or one that is repeated at least five times. Among these repeats, 61 tandem repeats are associated with 47 *M. bovis* lipoproteins, and 16 repeats are located in promoter regions. Over half of the lipoproteins, including the major surface lipoprotein P48, and six variable surface lipoproteins contain tandem repeats in the coding sequence or the promoter region and may cause protein sequence changes or expression changes in different growth phases. Two obvious examples are *MMB_0316* and *MMB_0434*. *MMB_0316* encodes the variable surface lipoprotein Y and has a 39-bp repeat unit with 9.7 copies found 98 bp downstream of its start codon. *MMB_0434* encodes a putative variable prolipoprotein, and it has four different tandem repeats with repeat units ranging from 10 to 69 bp in its coding sequence and 44 base repeats located in the region from −67 to −22 upstream of its start codon. The tandem repeats in the coding region cause a frameshift during replication, thus helping the antigen escape an immune response during the infection process. The tandem repeats in the promoter region can cause a change in the level of gene expression and affect antigen production. Both the promoter and the coding sequence are regulated by tandem repeats, indicating that MMB_0434 is an important *M. bovis* antigen.

In addition to the lipoproteins, 12 extracellular and 18 transmembrane proteins are also affected by tandem repeats. For example, there are two tandem repeats at the 3′ end of *MMB_0258*, which encodes for a hemolysin-related protein.

It is noteworthy that several tandem repeats are located adjacent to restriction-modification (RM) systems, which perhaps cause RM gene degeneration or phase-specific expression. We also observed a 60-bp long tandem repeat at the 5′-end of the 50S ribosomal protein L4 gene, which is disrupted in *M. bovis* and may be due to the tandem repeats.

### Replication origin and DNA replication

The *oriC* region is frequently located within the *rnpA–rmpH–dnaA–dnaN–recF–gyrB* gene cluster and is usually next to the *dnaA* gene [21]. However, in *M. bovis*, *recF* is not found, and the other five genes are located in three different loci. *rnpA-rmpH* is located ∼70 kb downstream of *dnaA-dnaN*, and *gyrB* is located ∼27 kb upstream of *dnaA*. The *oriC* region is also characterized by DnaA box motifs of which the consensus sequence is 5′-TTATCCACA-3′ [22]. We identified two perfect DnaA boxes in the area surrounding the *dnaA* gene, one is 60 bp upstream of *dnaA*, and the other is located 12,924 bp downstream of *dnaA*. By examining the intergenic region between *dnaA* and *dnaN*, we also found a noncanonical DnaA box (consensus sequence: 5′-TTTTAAAAA-3′) that has been reported in *Borrelia burgdorferi* [23]. The prescence of DnaA boxes near *dnaA* indicates that this region is a putative replication origin; thus, we designated the first *dnaA* base as the first base of the *M. bovis* genome. Although we found no features of a terminus of replication, a GC skew inversion was found at position 563,113 that could be considered the terminus of replication.

We found 20 DNA replication proteins in the *M. bovis* genome (**Table S4**). The central enzyme, the DNA polymerase III holoenzyme, comprises six genes that separately encode the subunits alpha (DnaE), beta (DnaN), delta (HolA), delta′ (HolB), gamma/tau (DnaX) and a Gram-positive type subunit alpha (PolC), which has been known to endow the strain with 3′ to 5′ exonuclease activity [24]. In addition to DNA polymerase III, four genes are also involved in DNA elongation including two RNaseH genes, one DNA ligase gene (*ligA*) and the truncated DNA polymerase I gene (*polA*). With the exception of *dnaA*, four additional genes were found to participate in DNA replication initiation. However, only one gene was identified as a DNA replication termination factor.

### Transcription and translation

Seventeen genes are predicted to be involved in transcription including four genes that encode the DNA-dependent RNA polymerase subunits (i.e., alpha, beta, beta′ and sigma) (**Table S5**). Elongation and transcription termination are regulated by three Nus factors, NusA, NusB and NusG, and one Gre factor, GreA. GreA could prevent transcription arrest, and NusA could induce transcription pausing or stimulate anti-termination together with NusB and NusG [25]. Only two transcription factors were found in the *M. bovis* genome, with one heat-inducible transcription repressor (MMB_0602, HrcA) likely providing negative regulation [26].

A total of 102 *M. bovis* genes are involved in translation including 47 ribosomal proteins, 20 tRNA synthetase genes and 10 translation factors (**Table S6**). Among these genes, the 50S ribosomal protein L4 is disrupted and phenylalanyl-tRNA synthetase is composed of two subunits, while glutaminyl-tRNA synthetase is absent. The translation factors include one ribosome-binding factor, three initiation factors, four elongation factors and two peptide chain release factors.

### Transporter and secretion systems

The *M. bovis* transporter system consists of 54 genes, which mainly constitute the ATP-binding cassette (ABC) transporter system and the phosphotransferase system (PTS) (**Table S7**). Two genes, *pts*I (MMB_0676) and *pts*H (MMB_0504), encode the PTS Enzyme I (EI) and phosphocarrier protein HPr and were identified together with an operon (MMB_0684 to MMB_0682) encoding ascorbate-specific IIA, IIB and IIC proteins, which comprise a PTS system for ascorbate absorbance. The other 49 genes encode components of the ABC transporter system including 22 ATP-binding proteins, 20 permease proteins and seven substrate-binding proteins. Thirty-one of the 49 genes encode seven complete ABC type transporter systems including two oligopeptide transport systems (MMB_0037 to MMB_0033; MMB_0105 to MMB_0109), two glycerol transport systems (MMB_0249 to MMB_0253 and MMB_0707 to MMB_0704), one sugar transport system (MMB_0017 to MMB_0021), one permidine/putrescine transport system and one alkylphosphonate transport system. Though complete ABC type transport systems were not found for the remaining 18 genes, several lipoproteins are adjacent to these genes, and these may work as substrate-binding proteins.

*M. bovis* encodes 13 proteins that are involved in protein secretion (**Table S8**), including SecA, SecD, SecE and SecY. Although secretion via the Sec pathway requires the presence of an N-terminal signal peptide on the secreted protein, signal peptidase (SPaseI) was absent in *M. bovis*. The channel-forming proteins SecF and SecG were also absent in the genome; consequently, the proteins are presumed to be exported through signal-recognition particle (SRP), which is composed of Ffh and FtsY. We also identified a protein (MMB_0050) belonging to the Type II secretion system.

### Metabolism

The *M. bovis* biosynthetic capacity is severely limited. The 6-phosphofructokinase gene of glycolysis (EMP cycle) is absent, and the genes involved in the TCA cycle and the oxidative phase of the pentose phosphate pathway are also missing. Most amino acids, purines, pyrimidines and cofactors cannot be *de novo* synthesized. [27]. During its intracellular lifestyle *M. bovis* obtains most of its nutrition from the host.

An extracellular cysteine protease (MMB_0708) was identified that may degrade host proteins into oligopeptides. Thus, two oligopeptide transport systems may import oligopeptides for further usage. Thirteen cytoplasmic peptidases were found in the genome (**Table S9**) that could degrade oligopeptides into amino acids to satisfy the *M. bovis* nitrogen requirement.

Genome analysis revealed that *M. bovis* uses glycerol instead of sugar as a carbon source. *M. bovis* assimilates glycerol through two glycerol ABC transport systems and one glycerol uptake facilitator protein (MMB_0301). Glycerol is phosphorylated by glycerol kinase (MMB_0302) to become glycerol 3-phosphate. Glycerol-3-phosphate dehydrogenase (MMB_0051) transforms glycerol 3-phosphate into glycerone phosphate, which is transformed into glyceraldehyde 3-phosphate by triosephosphate isomerase (MMB_0553). Glyceraldehyde 3-phosphate is involved in the EMP pathway and, finally, transforms into pyruvate. In this process, one molecule of ATP is consumed, and two molecules of ATP are produced. Thus, substrate-level phosphorylation is the major energy generating pathway.

Glycerol may be derived from degraded host lipids, such as serum triglycerides. It is well known that *Mycoplasma* has lipase activity [28]. In the *M. bovis* genome, six esterase/lipase genes were found, and these may encode proteins that can guarantee a supply of glycerol in the intracellular lifestyle of the organism.

Pyruvate is transformed into acetyl-CoA by the pyruvate dehydrogenase complex (MMB_0096 to MMB_0100) and into lactate by D-lactate dehydrogenase (MMB_01520) and L-lactate dehydrogenase (MMB_0526). Acetyl-CoA is transformed into acetyl phosphate by phosphate acetyltransferase (MMB_0142), and acetate kinase (MMB_0143) transforms acetyl phosphate into acetate, which results in the production of another ATP molecule. Because *M. bovis* has lost most of its biosynthesis capacity, energy production through acetyl-CoA may be another important energy generating pathway.

### Oxygen Stress

We did not identify superoxide dismutases (SODs), catalase, glutathione peroxidase or glutathione reductases, which could scavenge superoxide radicals (O_2_^2−^) and hydrogen peroxide (H_2_O_2_) damage in *M. bovis*. The main mechanisms to defend against oxygen stress in *M. bovis*, include a thioredoxin system, which includes two thioredoxins and a thioredoxin reductase, a thiol peroxidase (MMB_0208) and a peptide methionine sulfoxide reductase (MMB_0449) (**Table S10**).

As previously reported [29], glycerol 3-phosphate oxidation may produce H_2_O_2_ and cause host cell damage. Although glycerophosphate oxidase (GlpO) was not found in *M. bovis*, a transmembrane glycerol-3-phosphate dehydrogenase (MMB_0051) may be substituted in the presence of O_2_. Thus, the released H_2_O_2_ could be considered a *M. bovis* virulence factor.

### The RM system

*M. bovis* contains two compete type I RM systems (**Table S11**). One comprises MMB_0610 to MMB_0612 and exhibits homology with the type I RM system of *M. agalactiae*; however, the ortholog of *M. agalactiae* HsdR (MMB_0610) is fragmented into pseudogenes. Another type I RM system (MMB_0227, MMB_0228 and MMB_0231) has no homologous proteins in *M. agalactiae*. Thus, *M. bovis* exhibits a stronger barrier for gene transfer than *M. agalactiae*. In addition, gene fragments of the Type III RM system methylase gene and the Type II RM system endonuclease subunit were found in the genome indicating that Type II and Type III RM systems once existed in the genome but are now lost.

### Virulence factors

Two islands that have a lower GC content were found in the genome (733,550 to 737,937 and 831,641 to 838,944) (**Table S12**), and they have a bias for binucleotides and an IS element at the end. The 4.4 kb island encoded one variable prolipoprotein with an IS*Mbov2* element at the 3′ end. The 7.3 kb island encodes two lipoproteins with and IS*Mbov4* element at the 5′ end. Homology search of the island sequences revealed IS*Mbov2* in *M. mycoides* (see below), indicating the possibility that IS elements are derived from genomic islands transfer. Although the detailed function of these genes remain elusive, the pathogenicity of the islands is essential for virulence in animal pathogens [30].

In addition, we identified eight variable surface lipoproteins displaying antigenic variation. Among them, MMB_0316, MMB_0419 and MMB_0431 were specific for *M. bovis*. Another four ORFs encoding lipoproteins P30, P40, P48 and P80 were also found in the genome (**Table 2**). In all, 45 lipoproteins were found in the genome. These lipoproteins may be associated with *M. bovis* virulence.

**Table 2:** Potential virulence factors in *M.bovis*.

Locus	Description
MMB_0010	lipoate-protein ligase A
MMB_0011	lipoate-protein ligase A
MMB_0062	lipoate-protein ligase A
MMB_0100	dihydrolipoamide dehydrogenase (E3 component ofpyruvate complex) pdhD
MMB_0127	oligoendopeptidase F (pepF)
MMB_0129	spermidine/putrescine ABC transporter permease (potB)
MMB_0167	major surface lipoprotein P48
MMB_0258	hemolysin-related protein
MMB_0271	Vpma-like, lipoprotein
MMB_0316	variable surface lipoprotein Y (vpmaY1)
MMB_0345	conserved hypothetical protein
MMB_0419	variable surface lipoprotein Y (vpmaY2)
MMB_0422	P30, lipoprotein
MMB_0431	variable surface lipoprotein A (vpmaX)
MMB_0485	putative variable surface prolipoprotein
MMB_0494	cation-transporting P-type ATPase
MMB_0495	ABC transporter, permease protein
MMB_0496	ABC transporter, ATP-binding protein
MMB_0540	P80, lipoprotein
MMB_0543	ABC transporter ATP-binding protein
MMB_0544	ABC transporter permease protein
MMB_0545	ABC transporter permease protein
MMB_0614	putative variable prolipoprotein
MMB_0636	5′nucleotidase
MMB_0664	ClpB
MMB_0665	N-terminal truncated GCATC–recognizing Type II restriction modification system (MmyCIII) endonuclease subunit
MMB_0666	truncated GCATC–recognizing Type II restriction modification system (MmyCIII) endonuclease subunit
MMB_0668	C-terminal truncated GCATC–recognizing Type II restriction modification system (MmyCIII) endonuclease subunit
MMB_0688	adenine-specific DNA-methyltransferase (dam)
MMB_0693	prolipoprotein Q (lppQ1)
MMB_0708	putative cysteine protease
MMB_0718	conserved hypothetical protein
MMB_0756	hypothetical protein
MMB_0794	prolipoprotein Q (lppQ2)
MMB_0798	cation-transporting P-ATPase (mgtA)
MMB_0799	ABC transporter ATP-binding protein
MMB_0800	ABC transporter permease protein

There is one hemolysin-related protein (MMB_0258) in the genome. It has been shown that hemolysins are toxic proteins that cause the lysis of erythrocytes by forming pores in their membranes [31]. Hence, the *M. bovis* hemolysin may be considered a *M. bovis* virulence factor.

Three lipoate protein ligase (LplA) genes (*MMB_0010, MMB_0011 and MMB_0062*) were identified in the *M. bovis* genome. LplA ligates lipoic acid from host cells to the E2 subunit of the pyruvate dehydrogenase enzyme (PDH) complex to produce E2-lipoamide, which plays a pivotal role in aerobic metabolism. Defective LplA specifically damages the growth of *L. monocytogenes* in the host cytosol and reduces virulence in animals 300-fold [32]. In addition, a mutant of dihydrolipoamide dehydrogenase, an E3 PDH component, has demonstrated significant attenuation of *M. gallisepticum in vivo* [33]. Thus, LplA and dihydrolipoamide dehydrogenase (MMB_0100) are considered *M. bovis* virulence factors.

The extracellular cysteine protease (MMB_0708) is also an important virulence factor that could directly degrade the extracellular matrix (ECM) proteins fibronectin and vitronectin or activate host matrix metalloproteases to degrade the extracellular matrix [34], which would cause tissue damage in the host.

*M. bovis* secretes a 5′-nucleotidase (MMB_0636) that could utilize various nucleotides, such as ATP or ADP, from the host as a substrate and enhance macrophage death [35].

ClpC is an ATPase that promotes the early escape from macrophage phagosomes [36] and is also required for cell adhesion and invasion [37]. Although the ClpC gene is not found in the genome, *M. bovis* encodes for ClpB (MMB_0664), which shows 50% identity with *Listeria monocytogenes* ClpC, which may be considered a virulence factor.

MMB_0798 encodes a cation-transporting P-ATPase (MgtA), which is responsible for magnesium uptake and is required for intracellular survival.

In the *M. bovis* genome, three clusters may encode ABC type transporters that are involved in iron uptake (MMB_494, MMB_495 and MMB_496; MMB_543, MMB_544 and MMB_545; MMB_0799 and MMB_0780). The iron uptake system has been considered a virulence factor and is necessary for the utilization of iron bound to transferrin or iron chelates [38].

In our study, we also found that MMB_0127, MMB_0129, MMB_0345, MMB_0718 and MMB_0756 had immunogenicity. *MMB_0129* encodes a transmembrane protein that functions as a spermidine/putrescine ABC transporter permease, and *MMB_0345* encodes an extracellular protein. While MMB_0127, MMB_0718 and MMB_0756 are all cytoplasmic proteins, *MMB_0127* encodes an oligoendopeptidase, and *MMB_0756* encodes a hypothetical protein specific for *M. bovis*.

Because the pathology of *M. bovis*-infected calves is similar to *M. mycoides*-induced CBPP, we compared the two genomes and found 20 horizontal transfer genes. Although some of these genes have no clear biological function (i.e., conserved hypothetical proteins), they may be related to virulence (**Table 2**). These genes will be further discussed in the ‘Horizontal Gene Transfer’ section.

### Evolutionary Position

**Figure 2:**
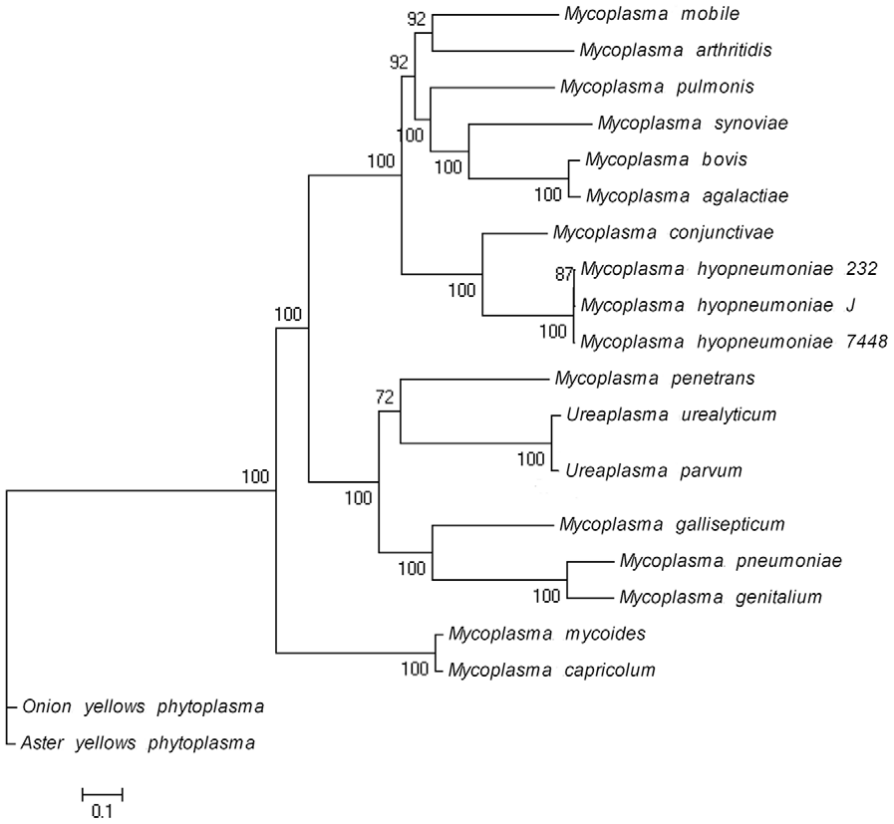
Phylogenetic tree of sequenced Mycoplasma. The tree was constructed using Maximum-likelihood method and rooted using Onion yellows phytoplasma and Aster yellows witches'-broom phytoplasma genome as the outgroup.

**Figure 3:**
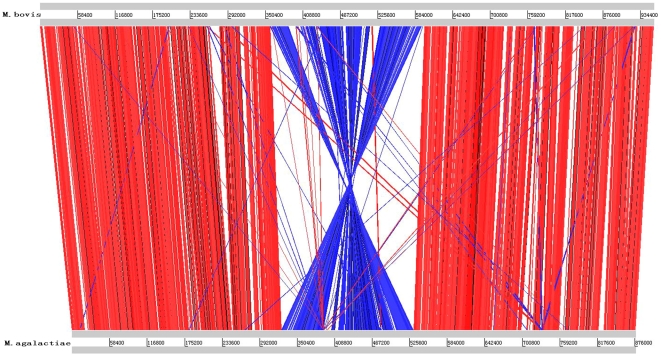
Comparison of genomic structure between *Mycoplasma bovis* Strain Hubei-1 and *Mycoplasma agalactiae* PG2. Red lines represented homologous sequences with same direction between the two genomes; blue lines represented homologous sequences with reversed direction between the two genomes.

We identified 150 orthologous genes between *M. bovis* and other *Mycoplasma* genomes. A phylogenetic tree based on the 150 concatenated orthologous proteins indicates a close relationship between *M. bovis* and *M. agalactiae* (Figure 2). The genomic structures of *M. bovis* and *M. agalactiae* show high synteny, with the exception of a 142-kb inversion in the middle of genome (Figure 3). This inversion may be induced by an IS element because an IS*Mbov1* element is adjacent to this region in *M. bovis*. The *M. bovis* genome is 70 kb larger than that of *M. agalactiae*. There are 682 *M. bovis* proteins (84.9%) with orthologs in *M. agalactiae*, and 82 proteins have orthologs in other *Mycoplasma* genomes. We found seven genes showing 97% similarity with the *Mycoplasma mycoides* genome but with no homology with other *Mycoplasma* genomes. These seven genes include four ISMbov2 transposases and three ISMbov3 transposases. Because *M. bovis* and *M. mycoids* are located in different phylogenetic branches (Figure 3) and have no genomic homology, it is not likely that they inherited *ISMbov2* and *ISMbov3* from a common ancestor. Instead, horizontal transfer of IS elements may occur when both *M. bovis* and *M. mycoids* infect the same bovine. Three proteins (MMB_0231, MMB_0429 and MMB_0670) are homologous to proteins outside of *Mycoplasma*, indicating a possible lateral gene transfer. Only 34 proteins have no match to known proteins and are considered *M. bovis* specific (**Table S13**).

### Pseudogenes, Paralogs and DNA repair systems

The *M. bovis* genome has 61 pseudogenes accounting for 7.6% of the total protein-coding genes (**Table S14**), yet the total size of these pseudogenes only occupy 3.4% of the genome. We identified 68 paralogous gene families in the *M. bovis genome* that comprise 218 genes (**Table S15**) and account for 27.1% of the *M. bovis* genes. The largest family consists of 13 ABC transporter ATP-binding proteins, and there are five families that comprise 35 transposases. Ten pseudogenes are included in the paralogous gene families, and their function may be complemented by their paralogs.

In the *M. bovis* genome, the pseudogenes mainly belong to the RM system (10 genes), which include the type I RM system DNA specificity subunit, the type II RM system endonuclease subunit and the type III RM system methylase subunit. It is noteworthy that an *ISMbov4* insertion split a type II RM system endonuclease gene into two pseudogenes (MMB_0666 and MMB_0668).

Two aminopeptidase genes were split into four pseudogenes in *M. bovis*; however, there are five additional complete aminopeptidase genes in the genome, which could complement the disruption of the two aminopeptidase genes. The same phenomenon is observed for esterase/lipase genes, where one esterase/lipase gene was split into two pseudogenes, yet six complete esterase/lipase genes guarantee efficient lipid metabolism.

With the exception of the DNA replication proteins, 22 genes are involved in DNA repair and recombination in *M. bovis* (**Table S16**); however, there are no mismatch-repair system (MutHLS) genes encoded in the genome. *M. bovis* DNA repair is mainly executed by recombinational repair, the SOS repair system, the nucleotide excision repair system and the base excision repair system. The recombination repair and SOS repair system is composed of RecA (MMB_0593), RecD (MMB_0804), RecR (MMB_0738), RuvA (MMB_0141), RuvB (MMB_0142) and the DNA-damage repair protein MucB (MMB_0075). The nucleotide excision repair system includes UvrA (MMB_0835), UvrB (MMB_0836), UvrC (MMB_0538) and DNA polymerase I (MMB_0169). The base excision repair system includes Ung (MMB_0069), MutM (MMB_0447) and Nfo (MMB_0535).

### Horizontal Gene Transfer

We found that 670 out of 803 *M. bovis* CDS have significant homology with *M. agalactiae* genes, which is the closest phylogenetically related *M. bovis* species. In addition, another 46 CDS showed homology to other *Mycoplasma* genomes located in the same group as *M. bovis*, while 54 CDS showed homology to the genomes outside of the group, reflecting possible horizontal gene transfer (**Table S17**). Of these horizontal transfer genes, 29 came from *M. mycoides*, a pathogen that causes CBPP. These 29 genes include four ISMbov2 transposases and three ISMbov3 transposases. While a homology search showed that ISMbov2 and ISMbov3 have no homologs in the *M. agalactiae* genome, there are homologs in *M. mycoides* that exhibit a 97% identity with the *M. mycoides* genome on the nucleotide level. This is direct evidence that horizontal transfer occurred between *M. bovis* and *M. mycoides*.

Because the pathological changes in the lungs of *M. bovis*-infected calves are sometimes similar to CBPP, we hypothesize that these 29 horizontal transfer genes are related to virulence. Among these 29 genes, two genes (MMB_0693 and MMB_0794) encode prolipoprotein Q, and four genes (MMB_0358, MMB_0434, MMB_0485 and MMB_0614) encode variable prolipoprotein. MMB_0434 is 58% identical to MSC_0364, and MMB_0358 (64% identity) and MMB_0485 (53% identity), two known prolipoprotein of *M. myvoides* associated with antigenic variation, both show homology to MSC_1005 [39].

Besides the 29 genes, MMB_0328, MMB_0427 and MMB_0429 encode lipoproteins and may also be considered potential virulence factors. Moreover, two CDS show homology to genomes outside of Mollicutes. The MMB_0488 gene encodes a protein that is similar to a *Janibacter* sp.HTCC2649 hypothetical protein, and MMB_0566 encodes a protein that is similar to a *Clostridium spiroforme* hypothetical protein.

### Comparison with *M. bovis* PG45

During the preparation of this manuscript, the *M. bovis* PG45 genome sequence was reported. The PG45 genome size is 1,003,404 bp, 50,290 bp longer than the Hubei-1 genome. Comparison of the two genomes revealed a 540-kb genome inversion (**Figure S1**). In PG45, the inversion (248,834 to 788,372) is located between two IS*Mbov3* elements, and thus, we could speculate that the inversion is caused by the combination of these IS elements. However, in *M. bovis* Hubei-1, ISM*bov1* is upstream of this inversion (200,618 to 757,702), and no IS element was identified on the other side. A 41-kb region (207,849 to 248,833) upstream of the PG45 inversion is absent in *M. bovis* Hubei-1, which may contribute to the genomic size difference between the two strains. This region contains an ICEB-2 element (Integrative Conjugal Element) that is 37,415 bp long, and several IS elements residing in ICEB-2 may cause its instability. This perhaps explains the absence of ICEB-2 in Hubei-1. Except for this inversion, the genomic content between the two genomes is conserved, and their sequence homology is 98.5%, with 62 identified insertions and 84 deletions (in *M. bovis* Hubei-1 (**Table S18**).

The largest insertion in Hubei-1 is 18.8 kb and encodes 13 proteins (MMB_0316-MMB_0328), including the variable surface lipoprotein Y and an IS*Mbov1* transposase. The existence of this fragment in Hubei-1 may be due to the transfer of IS*Mbov*1. Another large insertion is 10.7 kb, which encodes nine proteins (MMB_0227 to MMB_235), including a complete type I RM system (**Table S11**). Moreover, 21 insertions were IS elements. For deletions, with the exception of the ICEB-2 element, two PG45 fragments of 17.8 kb and 10.2 kb were also lost in the Hubei-1 genome. The 17.8-kb fragment encodes 13 variable surface lipoproteins and one transposase (MBOVPG45_0806 to MBOVPG45_0821), implicating an IS element role in this fragment transfer. The 10.2-kb fragment encodes five proteins (MBOVPG45_0616 to MBOVPG45_0621), including a transposase, a site-specific recombinase and three type I RM system enzymes. Although both the 10.7-kb insertion and the 10.2-kb deletion encode type I RM systems and occur in the same genome loci (**Table S18**), they have no homology at the nucleotide level. In other words, Hubei-1 and PG45 both possess a complete type I RM system, but they encode proteins with low similarity, which may endow the strains with different abilities to resist gene transfer. In addition, 32 deletions are in IS elements.

Homology searching between the Hubei-1 and PG45 genomes found 662 orthologs, which may be considered the ‘core genome’ of *M. bovis* (**Table S19**). As we can see from COG classification (**Table 1**), most functional category genes were shared by the two genomes, meaning that the two strains fundamentally possess the same genetic background for metabolism and growth. The only exception is the ‘DNA replication, recombination and repair’ category because 25 transposases in the Hubei-1 genome and 34 transposases in the PG45 genome are classified into this category, and only a few of these transposase genes have orthologs. Moreover, 51 Hubei-1 genes and 46 PG45 genes are unique for each genome and may represent a ‘specific genome’ of Hubei-1 and PG45, respectively (**Table S19**). The specific genes of the PG45 genome are mainly composed of hypothetical proteins and variable surface lipoproteins, which are located in the ICEB-2 element. Because the ICEB-2 element is absent in Hubei-1, the Hubei-1 specific genes include other lipoproteins, such as P40. It is noteworthy that two type II restriction endonucleases were among the PG45 specific genes, and several truncated type III RM system methylases were found among the Hubei-1 specific genes. The difference in the specific RM system genes may hint that the two strains have different capacities for undergoing lateral gene transfer.

## Discussion

While the *M. bovis* strain Hubei-1 genome encodes 73 lipoproteins and 36 secreted proteins, the SpaseI gene is not found in the genome; however, a lipoprotein signal peptidase gene (*lsp*) is predicted instead. In some *Mycoplasma* species, such as *M. conjunctivae*, *M. hyopneumoniae*, *M. pulmonis* and *M. synoviae*, both the signal peptidase I gene and the lipoprotein signal peptidase genes exist. Thus, extracellular protein secretion in *M. bovis* may have a different mechanism.

IS elements, which are usually associated with lateral gene transfer and genomic evolution, are abundant in the *M. bovis genome* (5.4%) and may cause genomic variation in different strains. We found two kinds of IS elements, IS*Mbov2* and IS*Mbov3*, in both the *M. bovis* and *M. mycoids* genomes. Because there is no genome homology between *M. bovis* and *M. mycoids*, the existence of these IS elements in both genomes is evidence of horizontal transfer between the genome.

*M. agalactiae* is the closest phylogenetically related *M. bovis* neighbor, and the two genomes exhibit high synteny in their genome structure, with only 15% of *M. bovis* genes lacking orthologs in *M. agalactiae*.

Over half of the lipoproteins and several extracellular and transmembrane proteins contain tandem repeats in their coding sequence or promoter regions. These phase-variable proteins exhibit protein sequence variation or expression level variation in different growth phases, and thus, they may change the antigen and *M. bovis* surface structure to help the organism evade the host immune response.

The *M. bovis* transporter system is mainly composed of ABC transport systems and PTS, which are in charge of oligopeptide, glycerol, sugar, alkylphosphonate and iron uptake.

As an intracellular pathogen, most of the biosynthesis pathways are incomplete in *M. bovis*. Glycerol, instead of sugar, is the *M. bovis* carbon source, which is transformed into glyceraldehyde 3-phosphate and participates in the EMP pathway to process pyruvate, with one molecule of ATP produced. Pyruvate is then transformed into acetate through two steps resulting in the production of another ATP molecule. Thus, the process of transforming glycerol into acetate produces two molecules of ATP, which may be an important energy generating pathway.

No mismatch-repair system (MutHLS) genes are encoded in the genome; thus, mutations may accumulate with strain replication and cause some genetic degeneration. The *M. bovis* pseudogenes mainly belong to the RM system, and the reduction in RM systems increase the possibility of horizontal gene transfer. The function of other pseudogenes may be complemented by their paralogs.

Because pathological changes in the lungs of *M. bovis*-infected calves are similar to CBPP, 29 *M. bovis* genes with homology to *M. myvoides* are also considered virulence factors. Moreover, 73 *M. bovis* lipoproteins could also be considered potential virulence factors, including eight variable surface lipoproteins, and the known virulence factors P30, P40, P48 and P80. Furthermore, our study validated that five proteins, including three cytoplasmic proteins, have immunogenicity. Other virulence factors include two pathogenicity islands, phase-variable proteins, a hemolysin-related protein, lipoate protein ligase (LplA), dihydrolipoamide dehydrogenase (the E3 component of PDH), extracellular cysteine protease, 5′-nucleotidase, ClpB, cation-transporting P-ATPase (MgtA) and ABC type transporters involved in iron uptake. Because glycerol 3-phosphate oxidation may produce H_2_O_2_ and cause host cell damage, the released H_2_O_2_ could also be considered a *M. bovis* virulence factor. These potential virulence factors could be used as candidates for drug therapy and vaccine design.

## Materials and Methods

### Bacterial growth and DNA extraction

For *Mycoplasma* growth, we used a modified ATCC 1699 broth (0.8 g glucose, 20% pig serum, 100 units of penicillin, and 0.05% acetic acid thallium). A commercial tissue genomic DNA extraction kit (Axygen, Inc., USA) was used to purify DNA.

### High-density pyrosequencing and sequence assembly of the genome

The complete genomic sequencing was conducted using a Roche GS FLX system [40]. A total of 85,820 reads totaling 34,770,094 bases (average read length: 405 bp), was obtained resulting in 38-fold genome coverage. Assembly was performed using the GS de novo Assembler software (http://www.454.com/) and produced 86 contigs ranging from 500 bp to 71,153 bp (the N50 contig size is 27,842 bp). The relationship of the contigs was determined by multiplex PCR [41]. Gaps were then filled in by sequencing the PCR products using ABI 3730xl capillary sequencers. Phred, Phrap and Consed software packages (http://www.genome.washington.edu) were used for final assembly and editing, and low quality regions of the genome were resequenced. The final sequencing accuracy was 99.99%.

### Genome annotation

Putative CDS were identified by Glimmer 3.02 [42] and ZCURVE 1.02 [43], and peptides shorter than 30 amino acids were eliminated. Insert sequences were first detected using the IS Finder database (http://www-is.biotoul.fr/is.html) using the default parameters and manual selection. Transfer RNA genes were predicted by tRNAScan-SE [44]. Functional annotation of CDS was performed by searching against an in-house developed Mollicutes protein database using BLASTP [45] and the CDD databases [46] by RPS-BLAST.

The metabolic pathways were constructed using the KEGG database [47]. Subcellular localization of the proteins was predicted by PSORTb (v2.0.1) [48], and lipoproteins were identified with LipoP 1.0 [49]. Pathogenicity islands were detected by IslandViewer [50]. Orthologs and paralogs were defined as proteins with greater than 30% similarity. Genome comparisons were performed using BLAST and displayed by the Artemis Comparison Tool (ACT) [51]. The genome atlas was drawn using GenomeViz1.1 [52].

### Phylogenetic tree construction

Orthologs of known Mycoplasma genomes were obtained from the MBGD database [53]. The *M. bovis* strain Hubei-1 phylogenetic position within the Mollicutes was determined based on 150 ortholog proteins. Concatenated protein sequences of 150 orthologous *mycoplasma* species proteins were first aligned using ClustalW [54], the conserved alignment blocks were then extracted by the Gblocks program [55]. A maximum likelihood tree was built with PHYML [56] with the following parameters: 100 replications for bootstrap analysis, “JTT” for the substitution model, “estimated” for the proportion of invariable sites, “estimated” for the gamma distribution parameters, “4” for the number of substitution categories, “yes” to optimize tree topology, and “BIONJ” for starting tree(s).

### Nucleotide sequence accession numbers

The complete genomic sequences of *M. bovis* strain Hubei-1 have been deposited in GenBank under accession number CP002513.

## Supporting Information

Figure S1 **Comparison of genomic structure between** ***Mycoplasma bovis*** **Strain Hubei-1 and** ***Mycoplasma bovis*** **PG45.** Red lines represented homologous sequences with same direction between the 2 genomes; blue lines represented homologous sequences with reversed direction between the 2 genomes.
(TIF)

Table S1 **Codon usage in** ***M.bovis*** **Hubei-1.**
(XLS)

Table S2 **IS elements distribution in** ***M.bovis***
(XLS)

Table S3 **Tandem repeats in** ***M.bovis***
(XLS)

Table S4 **Predicted genes involved in DNA replication.**
(XLS)

Table S5 **Genes involved in transcription**
(XLS)

Table S6 **Translation factors in** ***M.bovis***
(XLS)

Table S7 **Transporters of** ***M.bovis***
(XLS)

Table S8 **Proteins involved in secretionary system**
(XLS)

Table S9 **Protease/Peptidase of** ***M.bovis***
(XLS)

Table S10 **Oxygen stress resistant genes**
(XLS)

Table S11 **Restriction-Modification system of** ***M.bovis***
(XLS)

Table S12 **Possible pathogenicity islands in** ***M.bovis***
(XLS)

Table S13 ***M.bovis*** **specific protein**
(XLS)

Table S14 **Pseudogenes in** ***M.bovis*** **genome**
(XLS)

Table S15 **Paralogs of** ***M.bovis*** **genome**
(XLS)

Table S16 **Predicted genes involved in DNA repair**
(XLS)

Table S17 **Possible horizontal transfer gene of** ***M.bovis***
(XLS)

Table S18 **Insertions and Deletions of** ***M.bovis*** **Hubei-1 relative to PG45**
(XLS)

Table S19 **Orthologs and specific genes of Hubei-1 and PG45 of** ***M.bovis***
(XLS)

## References

[1] Adegboye DS, Halbur PG, Nutsch RG, Kadlec R, Rosenbuch RF (1996). *Mycoplasma bovis*-associated pneumonia and arthritis complicated with pyogranulomatous tenosynovitis in calves.. JAVMA.

[2] Walz PH, Mullaney TP, Render JA (1997). Otitis media in preweaned holstein dairy calves in Michigan due to *Mycoplasma bovis*.. J Vet Diagn Invest.

[3] Pfutzner H, Saches K (1996). *Mycoplasma bovis* as an agent of matitis, pneumonia, arthritis and genital disorders in cattle.. Rev Sci Tech Off Int Epiz.

[4] Maunsell FP, Donovan GA (2009). *Mycoplasma bovis* infection in young calves.. Vet Clin Food Anim.

[5] Robin P, Alberti A, Oittau M, Chessa B, Miciletta M (2005). Genetic and antigenic characterization of the surface lipoprotein P48 of *Mycoplasma bovis*.. Vet Micro.

[6] Thomas A, Ball H, Dizzier I, Trolin A, Bell C (2002). Isolation of mycoplasma species from the lower respiratory tract of healthy cattle and cattle with respiratory disease in Belgium.. Vet Rec.

[7] Milles H, Lesser W, Sears P (1992). The economic implication of bioengineered mastitis control.. J Dairy Sci.

[8] Maunsell FP, Donovan GA, Risco C, Brown MB (2009). Field evaluation of a Mycoplasma bovis bacterin in young dairy calves.. Vaccine.

[9] Caswell JL, Archambault M (2007). Mycoplasma bovis pneumonia in cattle.. Anim Health Res Rev.

[10] Robino P, Alberti A, Pittau M, Chessa B, Miciletta M (2005). Genetic and antigenic characterization of the surface lipoprotein P48 of Mycoplasma bovis.. Vet Microbiol.

[11] Foddai A, Idini G, Fusco M, Rosa N, de la Fe C (2005). Rapid differential diagnosis of Mycoplasma agalactiae and Mycoplasma bovis based on a multiplex-PCR and a PCR-RFLP.. Mol Cell Probes.

[12] Thomas A, Linden A, Mainil J, Dizier I, Baseman JB (2004). The p40* adhesin pseudogene of Mycoplasma bovis.. Vet Microbiol.

[13] Lysnyansky I, Yogev D, Levisohn S (2008). Molecular characterization of the Mycoplasma bovis p68 gene, encoding a basic membrane protein with homology to P48 of Mycoplasma agalactiae.. FEMS Microbiol Lett.

[14] Lysnyansky I, Sachse K, Rosenbusch R, Levisohn S, Yogev D (1999). The vsp locus of Mycoplasma bovis: gene organization and structural features.. J Bacteriol.

[15] Poumarat F, Perrin B, Longchambon D (1991). Identification of ruminant mycoplasmas by dot immunobinding on membrane filtration (MF dot).. Vet Micro.

[16] Hotzel H, Sachse K, Pfützner H (1996). Rapid detection of Mycoplasma bovis in milk samples and nasal swabs using the polymerase chain reaction.. J Appl Bacteriol.

[17] Bhugra B, Dybvig K (1993). Identification and characterization of IS1138, a transposable element from Mycoplasma pulmonis that belongs to the IS3 family.. Mol Microbiol.

[18] Dybvig K, Zuhua C, Lao P, Jordan DS, French CT (2008). Genome of Mycoplasma arthritidis.. Infect Immun.

[19] Chambaud I, Heilig R, Ferris S, Barbe V, Samson D (2001). The complete genome sequence of the murine respiratory pathogen Mycoplasma pulmonis.. Nucleic Acids Res.

[20] Neyrolles O, Chambaud I, Ferris S, Prevost MC, Sasaki T (1999). Phase variations of the Mycoplasma penetrans main surface lipoprotein increase antigenic diversity.. Infect Immun.

[21] Mackiewicz P, Zakrzewska-Czerwinska J, Zawilak A, Dudek MR (2004). Cebrat S. Where does bacterial replication start? Rules for predicting the oriC region.. Nucleic Acids Res.

[22] Fujikawa N, Kurumizaka H, Nureki O, Terada T, Shirouzu M (2003). Structural basis of replication origin recognition by the DnaA protein.. Nucleic Acids Res.

[23] Picardeau M, Lobry JR, Hinnebusch BJ (1999). Physical mapping of an origin of bidirectional replication at the centre of the Borrelia burgdorferi linear chromosome.. Mol Microbiol.

[24] Himmelreich R, Hilbert H, Plagens H, Pirkl E, Li BC (1996). Complete sequence analysis of the genome of th7e bacterium Mycoplasma pneumoniae.. Nucleic Acids Res.

[25] Borukhov S, Lee J, Laptenko O (2005). Bacterial transcription elongation factors: new insights into molecular mechanism of action.. Mol Microbiol.

[26] Chang LJ, Chen WH, Minion FC, Shiuan D (2008). Mycoplasmas regulate the expression of heat-shock protein genes through CIRCE-HrcA interactions.. Biochem Biophys Res Commun.

[27] van der Merwe J, Prysliak T, Perez-Casal J (2010). Invasion of bovine peripheral blood mononuclear cells and erythrocytes by Mycoplasma bovis.. Infect Immun.

[28] ROTTEM S, RAZIN S (1964). LIPASE ACTIVITY OF MYCOPLASMA.. J Gen Microbiol.

[29] Pilo P, Vilei EM, Peterhans E, Bonvin-Klotz L, Stoffel MH (2005). A metabolic enzyme as a primary virulence factor of Mycoplasma mycoides subsp. mycoides small colony.. J Bacteriol.

[30] Schmidt H, Hensel M (2004). Pathogenicity islands in bacterial pathogenesis.. Clin Microbiol Rev.

[31] Goebel W, Chakraborty T, Kreft J (1988). Bacterial hemolysins as virulence factors.. Antonie Van Leeuwenhoek.

[32] O'Riordan M, Moors MA, Portnoy DA (2003). Listeria intracellular growth and virulence require host-derived lipoic acid.. Science.

[33] Gates AE, Frasca S, Nyaoke A, Gorton TS, Silbart LK (2008). Comparative assessment of a metabolically attenuated Mycoplasma gallisepticum mutant as a live vaccine for the prevention of avian respiratory mycoplasmosis.. Vaccine.

[34] Burns EH, Marciel AM, Musser JM (1996). Activation of a 66-kilodalton human endothelial cell matrix metalloprotease by Streptococcus pyogenes extracellular cysteine protease.. Infect Immun.

[35] Kirillicheva GB, Kudriavtsev LIu, Belaia IuA, Tumanian MA (1987). [Functional macrophage activity in infection with virulent and avirulent strains of the dysentery microbe].. Zh Mikrobiol Epidemiol Immunobiol.

[36] Rouquette C, de Chastellier C, Nair S, Berche P (1998). The ClpC ATPase of Listeria monocytogenes is a general stress protein required for virulence and promoting early bacterial escape from the phagosome of macrophages.. Mol Microbiol.

[37] Nair S, Milohanic E, Berche P (2000). *ClpC ATPase is required for cell adhesion and* invasion of Listeria monocytogenes.. Infect Immun.

[38] Sanders JD, Cope LD, Hansen EJ (1994). Identification of a locus involved in the utilization of iron by Haemophilus influenzae.. Infect Immun.

[39] Westberg J, Persson A, Holmberg A, Goesmann A, Lundeberg J (2004). The genome sequence of Mycoplasma mycoides subsp. mycoides SC type strain PG1T, the causative agent of contagious bovine pleuropneumonia (CBPP).. Genome Res.

[40] Margulies M, Egholm M, Altman WE, Attiya S, Bader JS (2005). Genome sequencing in microfabricated high-density picolitre reactors.. Nature.

[41] Tettelin H, Radune D, Kasif S, Khouri H, Salzberg SL (1999). Optimized multiplex PCR: efficiently closing a whole-genome shotgun sequencing project.. Genomics.

[42] Delcher AL, Harmon D, Kasif S, White O, Salzberg SL (1999). Improved microbial gene identification with GLIMMER.. Nucleic Acids Res.

[43] Guo FB, Ou HY, Zhang CT (2003). ZCURVE: a new system for recognizing protein-coding genes in bacterial and archaeal genomes.. Nucleic Acids Res.

[44] Lowe TM, Eddy SR (1997). tRNAscan-SE: a program for improved detection of transfer RNA genes in genomic sequence.. Nucleic Acids Res.

[45] Altschul SF, Madden TL, Schäffer AA, Zhang J, Zhang Z (1997). Gapped BLAST and PSI-BLAST: a new generation of protein database search programs.. Nucleic Acids Res.

[46] Marchler-Bauer A, Anderson JB, Derbyshire MK, DeWeese-Scott C, Gonzales NR (2007). CDD: a conserved domain database for interactive domain family analysis.. Nucleic Acids Res.

[47] Kanehisa M, Goto S, Kawashima S, Okuno Y, Hattori M (2004). The KEGG resource for deciphering the genome.. Nucleic Acids Res.

[48] Gardy JL, Laird MR, Chen F, Rey S, Walsh CJ (2005). PSORTb v.2.0: expanded prediction of bacterial protein subcellular localization and insights gained from comparative proteome analysis.. Bioinformatics.

[49] Juncker AS, Willenbrock H, Von Heijne G, Brunak S, Nielsen H (2003). Prediction of lipoprotein signal peptides in Gram-negative bacteria.. Protein Sci.

[50] Langille MG, Brinkman FS (2009). IslandViewer: an integrated interface for computational identification and visualization of genomic islands.. Bioinformatics.

[51] Carver TJ, Rutherford KM, Berriman M, Rajandream MA, Barrell BG, Parkhill J (2005). Bioinformatics.

[52] Ghai R, Hain T, Chakraborty T (2004). GenomeViz: visualizing microbial genomes.. BMC Bioinformatics.

[53] Uchiyama I (2003). MBGD: microbial genome database for comparative analysis.. Nucleic Acids Res.

[54] Thompson JD, Higgins DG, Gibson TJ (1994). CLUSTAL W: improving the sensitivity of progressive multiple sequence alignment through sequence weighting, position-specific gap penalties and weight matrix choice.. Nucleic Acids Res.

[55] Castresana J (2000). Selection of conserved blocks from multiple alignments for their use in phylogenetic analysis.. Mol Biol Evol.

[56] Guindon S, Gascuel O (2003). A simple, fast, and accurate algorithm to estimate large phylogenies by maximum likelihood.. Syst Biol.

