# Supplementary figures and images for: The Complete Genome Sequence of Mycoplasma bovis Strain Hubei-1
Source: PLoS One. 2011 Jun 22;6(6):e20999. doi: 10.1371/journal.pone.0020999 (PMC3120828; doi:10.1371/journal.pone.0020999)

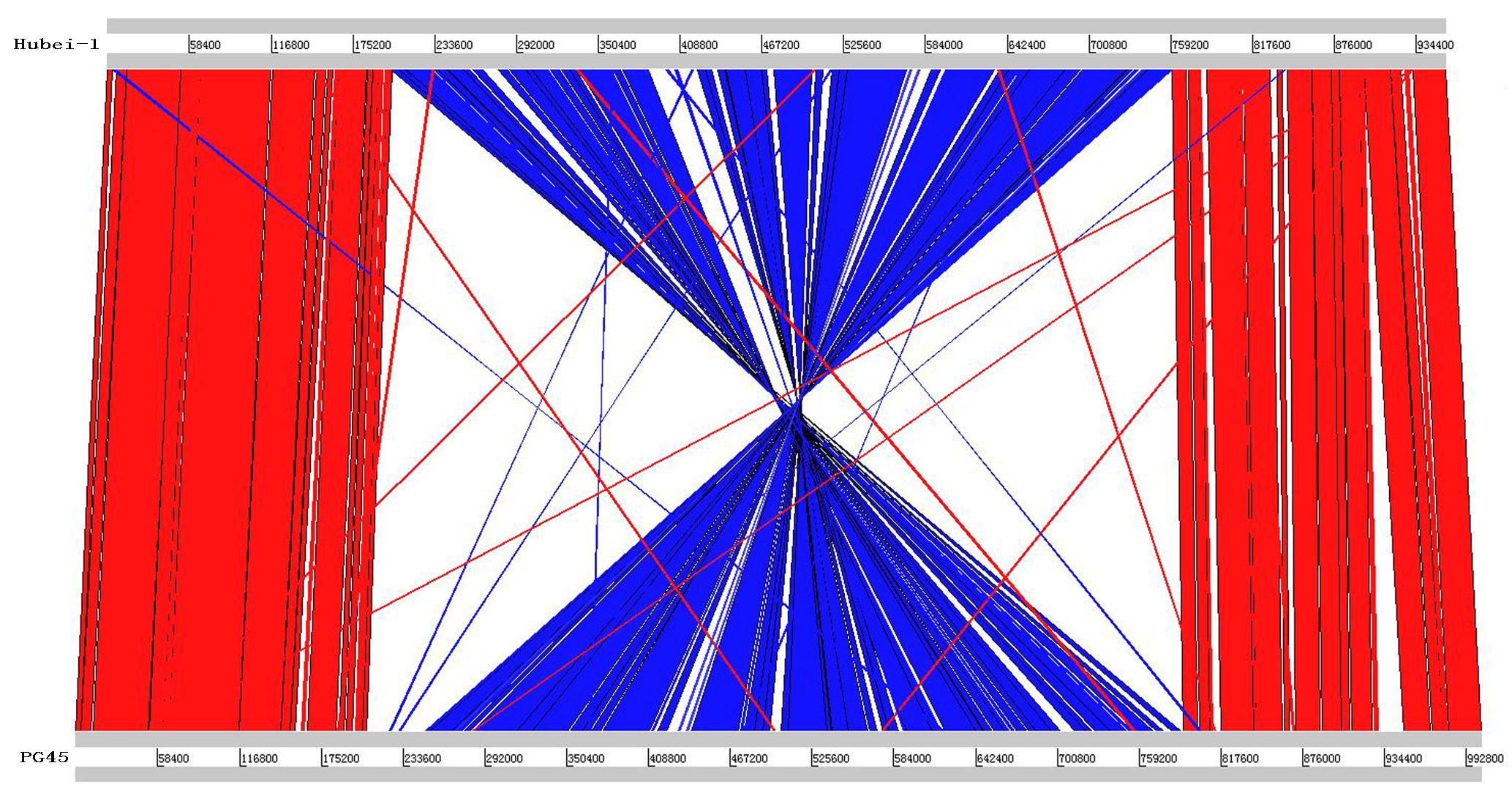

Supplement: Figure S1 — Comparison of genomic structure between Mycoplasma bovis Strain Hubei-1 and Mycoplasma bovis PG45. Red lines represented homologous sequences with same direction between the 2 genomes; blue lines represented homologous sequences with reversed direction between the 2 genomes. (TIF) [file pone.0020999.s001.tif]
